# Enhancement Strategy for Protocatechuic Acid Production Using *Corynebacterium glutamicum* with Focus on Continuous Fermentation Scale-Up and Cytotoxicity Management

**DOI:** 10.3390/ijms26010396

**Published:** 2025-01-05

**Authors:** Jiwoon Chung, Wooshik Shin, Chulhwan Park, Jaehoon Cho

**Affiliations:** 1 Department of Chemical Engineering, Kwangwoon University, 20, Gwangun-ro, Nowon-gu, Seoul 01897, Republic of Korea; wjdwldns1324@naver.com; 2 Low-Carbon Transition R&D Department, Korea Institute of Industrial Technology (KITECH), 89, Yangdaegiro-gil, Ipjang-myeon, Seobuk-gu, Cheonan-si 31056, Republic of Korea; 3 Regional Industrial Innovation Department, Korea Industrial Technology (KITECH), 89, Yangdaegiro-gil, Ipjang-myeon, Seobuk-gu, Cheonan-si 31056, Republic of Korea; sws@kitech.re.kr

**Keywords:** protocatechuate, *Corynebacterium glutamicum*, statistical medium optimization, continuous cultivation, cytotoxicity management, protocatechuic acid

## Abstract

Protocatechuate acid (PCA) is a phenolic acid naturally synthesized by various organisms. Protocatechuic acid is synthesized by plants for physiological, metabolic functions, and self-defense, but extraction from plants is less efficient compared to the microbial culture process. The microbial synthesis of protocatechuic acid is sustainable and, due to its high yield, can save energy consumption when producing the same amount. To enhance PCA production using *Corynebacterium glutamicum*, a statistical optimization of the production medium was performed using full factorial design, the steepest ascent method, and the response surface method. The optimized production medium enabled a PCA production of over 5 g/L in a 72 h batch culture. However, PCA cytotoxicity affected the strain growth and PCA production rate, with an inhibitory concentration of approximately 5 g/L in the fermentation broth. Finally, continuous fermentation was operated for 150 h in the steady-state mode, maintaining the concentration of PCA below 5 g/L. The optimization method established in this study successfully increased PCA production levels, and the findings presented herein are anticipated to contribute to the industrialization of PCA production using *C. glutamicum.*

## 1. Introduction

Protocatechuic acid (PCA; 3,4-dihydrobenzoic acid) is a phenolic compound commonly found in over 500 plant and microbial species [1,2,3,4,5]. PCA is a precursor of industrially significant aromatic compounds such as vanillin and cis-cis muconate [6,7,8]. PCA possesses excellent antioxidant activity, which is associated with its anticancer efficacy [9]. Especially, PCA anticancer activities have been demonstrated against human cancer cell lines such as MCF-7, A549, HepG2, HeLa, and LNCaP [10]. High production expenses and limited production yields are the complications associated with PCA [11,12,13].

In the past, protocatechuic acid was produced through extraction from plants or chemical synthesis. However, the extraction method from plants requires time-consuming processes such as cultivation, harvesting, and extraction of raw plant material. Specifically, the previous research involving the extraction of protocatechuic acid from *Scutellaria barbata D. Don* using supercritical carbon dioxide yielded approximately 64 μg/g, demonstrating a lower energy efficiency and production yield compared to microbial production methods [14]. Currently, various studies are being conducted to produce high-value-added substances using different biomass sources [15]. Several studies have been conducted on microbial production to enhance the PCA yield, and its synthesis has been attempted using microorganisms such as *Escherichia coli* [16], *Pseudomonas putida* [17,18], and *Corynebacterium glutamicum* [19,20]. *C. glutamicum* strains are used industrially to produce organic acids, amino acids, and nucleotides and are generally recognized as safe microorganisms [21,22,23,24,25,26]. *C. glutamicum* maintains its growth rate even at concentrations that inhibit aromatic compound growth in other strains [27,28,29,30,31,32]. However, 1.5 g/L of PCA restricts the growth of *E. coli* [12] because of the hydrophobic characteristic of aromatic compounds, such as PCA, which can accumulate in phospholipids within the membranes and cause membrane instability [8,12,19].

The enzyme 3-dehydroshikimate dehydrogenase of the PCA production pathway affects PCA productivity by feedback inhibition, which can be reduced by PCA with a Ki of approximately 0.38 mM and a Ki’ of approximately 0.96 mM through competitive and non-competitive inhibition mechanisms [20]. In addition, *P. putida* strains with similar metabolic pathways show feedback inhibition of aromatic amino acids on intermediate enzymes in the PCA production pathway, where conversion of erythrose-4-prosphate and phosphoenolpyruvate to 3-deoxy-D-arabino-heptulosonic acid 7-phosphate affects the production rates [17].

In this study, we employed process optimization to overcome these limitations and enhance PCA production efficiency. Improving the efficiency of microbial production processes can reduce the consumption of energy and resources required for operation, thereby contributing to environmental protection. The optimization of the fermentation process was conducted to minimize substrate consumption and overcome production concentration limitations, thereby reducing the environmental impact of the fermentation process itself. The strain utilized in PCA production, *C. glutamicum* AK103, was engineered: the *pcaGH* gene, which encodes the enzyme that converts PCA to catechol, and the *aroE* gene, which encodes the enzyme that converts the PCA precursor, 3-dehydroshikimate (DHS), to shikimate, were removed [30,33]. These modifications allow the strain to accumulate PCA. Deletion of the *aroE* gene in the AK103 strain blocks the biosynthetic pathway of aromatic amino acids, resulting in feedback inhibition of DHAP synthase [17,30]. However, supplying aromatic amino acids to the culture medium is problematic.

To solve this problem, we optimized the concentration of yeast extract that supplied aromatic amino acids to the initial PCA production medium. We further used statistical optimization methods, such as a full factorial design, steepest ascent method (SAM), and response surface method (RSM), to adjust the medium composition. Statistical optimization methods have been utilized in various research fields, such as chemical engineering, biotechnology, bioconversion, and optimization of the production and extraction of biomass-derived resources [21,34,35,36,37,38]. The three statistical media optimization methods presented were used for process development or bio-based production, and the experiments designed using the program were used to identify the interactions between media components and the product and the optimal point. We also established the conditions for continuous culture of *C. glutamicum* through preliminary experiments, thereby overcoming limitations in the maximum concentration of the product. The optimization methods applied to the protocatechuic acid fermentation process can reduce the energy consumed per unit of protocatechuic acid produced.

## 2. Results and Discussion

### 2.1. Effects of Yeast Extract as a Nitrogen Source in Production Medium

**Figure 1:**
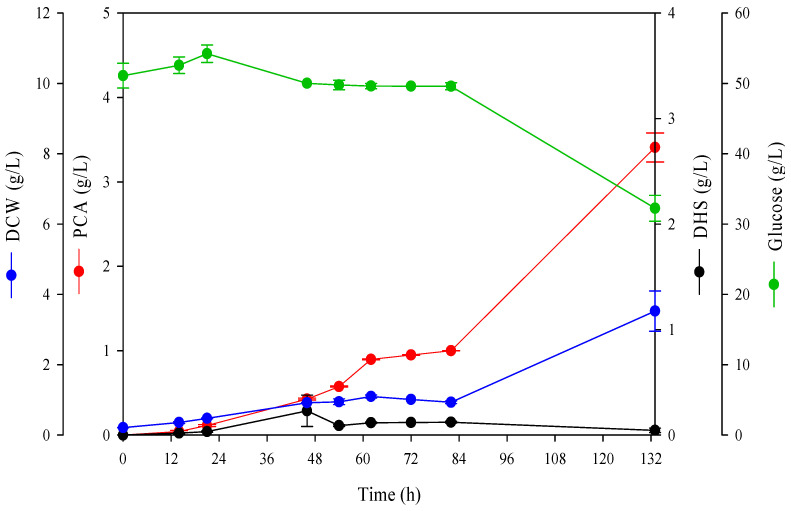
Effect of yeast extract concentration on PCA production during fermentation in a 5 L fermenter. The fermentation was conducted for 80 h in fed-batch culture by *C. glutamicum* AK103 with CPM_1. A total of 100 g/L of dissolved yeast extract was pulse-fed for 82 h. Error bars represent the standard deviation of two independent experiments. DCW, dry cell weight; PCA, protocatechuic acid; DHS, dehyroshikimic acid.

To optimize the 5 L lab-scale fermentation, experiments were conducted using the previously developed *Corynebacterium glutamicum* AK103 strain as the parent strain [33]. The *aroE* gene was knocked out in the AK103 strain to enable the biosynthesis of PCA using glucose as the carbon source. The removal of *aroE* resulted in no aromatic amino acid biosynthesis in the AK103 cells. During 80 h of fermentation, PCA production reached approximately 1 g/L, and the PCA precursor, 3-dehydroshikimic acid (DHS), was produced at 0.13 g/L (Figure 1). The DCW increased steadily up to 60 h of incubation, being approximately 4.2 g/L at 60 h, and then decreased slightly to 3.67 g/L at 80 h of incubation, which was confirmed to persist until 132 h, when the fermentation was completed (Figure 1). In contrast, the initially supplied glucose was utilized in small amounts, with approximately 5 g/L consumed after 80 h of fermentation, indicating low glucose utilization by the AK103 strain. A DCW of 1 g/L was assumed to result from the slow growth rate, and the low yeast extract concentration provided an insufficient supply of amino acids for cell growth [33]. It can also be inferred that the low PCA production levels resulted from the low glucose uptake. In the AK103 strain, the intermediate genes of the metabolic pathway required for the synthesis of aromatic amino acids were also removed [33]. To further confirm the effect of a sufficient supply of aromatic amino acids on PCA production, 100 g/L of dissolved yeast extract was pulse-fed at 82 h, additionally, in the same fermenter (Figure 1). The results showed that the PCA concentration increased to 3.41 g/L by the end of the fermentation period, and the glucose consumption was approximately 15 g/L. This demonstrated that an additional supply of yeast extract in the AK103 strain promoted PCA production. As our experiments demonstrated the effect of yeast extract on PCA production in CPM_1, we conducted experiments to optimize the production medium containing the yeast extract.

### 2.2. Statistical Optimization of the Production Medium

#### 2.2.1. Factorial Design

Using *C. glutamicum* AK103 strains, we optimized the medium for efficient PCA production. Based on the above results, it was estimated that 2 g/L of yeast extract in the CPM_1 medium was a limiting substrate for cell growth and PCA production (Figure 1). Yeast extract, as a complex nitrogen source, is characterized by its typically high aromatic amino acid content [39,40]. Flask cultivation experiments with AK103 across various yeast extract concentrations demonstrated that PCA productivity decreased when the yeast extract levels exceeded 22 g/L (Figure S1), indicating the presence of yeast extract concentrations beyond the optimal range in the culture medium. We optimized the yeast extract concentration at the flask scale and observed an average production of the highest PCA of 2.48 g/L in a production medium containing 18 g/L of yeast extract (Figure S1). The results showed an approximately 17-fold increase in production capacity compared to a previous production medium containing 2 g/L of yeast extract (Figure S1). Therefore, for the next step, the concentration of the yeast extract in the CPM_1 medium was adjusted to 18 g/L. Given that the modifications in yeast extract composition alone resulted in increased PCA concentrations, we hypothesized that implementing statistical media optimization methods to understand the medium component interactions could lead to further enhancement of productivity. We used a fractional factorial design (FFD) to minimize the number of experiments. The experimental design is shown in Tables S1 and S2. Analysis of variance (ANOVA) was performed using the statistical program Design Expert 12, and the *p*-value was significant at 0.0128 (Table 1). This indicates that the experimental results were highly reliable, with a validity of >98%. The equation derived from the ANOVA results was used to evaluate the association between the factors and PCA productivity (Table 1). The ANOVA results showed that, in the current medium composition, urea had a significant effect on PCA productivity compared to other components. Contribution analysis based on the ANOVA data revealed the following individual effects of media components: glucose (2.01%), urea (70.14%), (NH_4_)_2_SO_4_ (0.32%), yeast extract (0.65%), KH_2_PO_4_ (0.04%), and MgSO_4_ (1.04%). The factors demonstrating the highest impact on PCA production were identified in descending order as urea, glucose, yeast extract, and MgSO_4_. In particular, the individual effect of urea exhibited a *p*-value below 0.0001, demonstrating its superior statistical significance in affecting PCA productivity compared to other components under the current medium formulation. The coefficient value of −1.31 suggested that decreasing the urea concentration would be beneficial. The coefficient of the yeast extract was −0.13, which indicated that it should be similarly reduced in the production medium to increase PCA productivity. Elevated urea concentrations can inhibit cellular growth through multiple mechanisms, including osmotic stress, pH perturbation, and increased energy expenditure due to enhanced nitrogen metabolism [39]. Analysis of the mean square and F-value for the yeast extract in the FFD experiments indicated that it was the third most significant contributor to PCA productivity within the established concentration range among the factors studied. DHAP synthase feedback inhibition exhibits high sensitivity to intracellular aromatic amino acid concentrations, representing an evolutionarily conserved regulatory mechanism [33]. The pronounced effect of yeast extract can be attributed to this stringent metabolic regulation, indicating that deviation from the optimal concentration ranges may result in the inhibition of PCA production. Interestingly, the use of high yeast extract concentrations in the FFD resulted in decreased effectiveness. Based on the FFD, we determined the major factors affecting PCA productivity and found that optimization should be aimed at reducing the composition of yeast extract and urea, which are nitrogen sources in the production medium. The final equation was as follows: PCA = 3.11 + 2.23A − 1.36B + 0.09C − 0.13D + 0.03E + 0.17F − 0.06AB + 0.23AC + 0.06AD − 013AD + 0.02AE − 0.23BC + 0.21BD + 0.06BE − 0.15BF − 0.04CD + 0.14CE − 0.04CF + 0.14DE − 0.36DF + 0.28EF.

#### 2.2.2. Response Surface Method (RSM)

**Figure 2:**
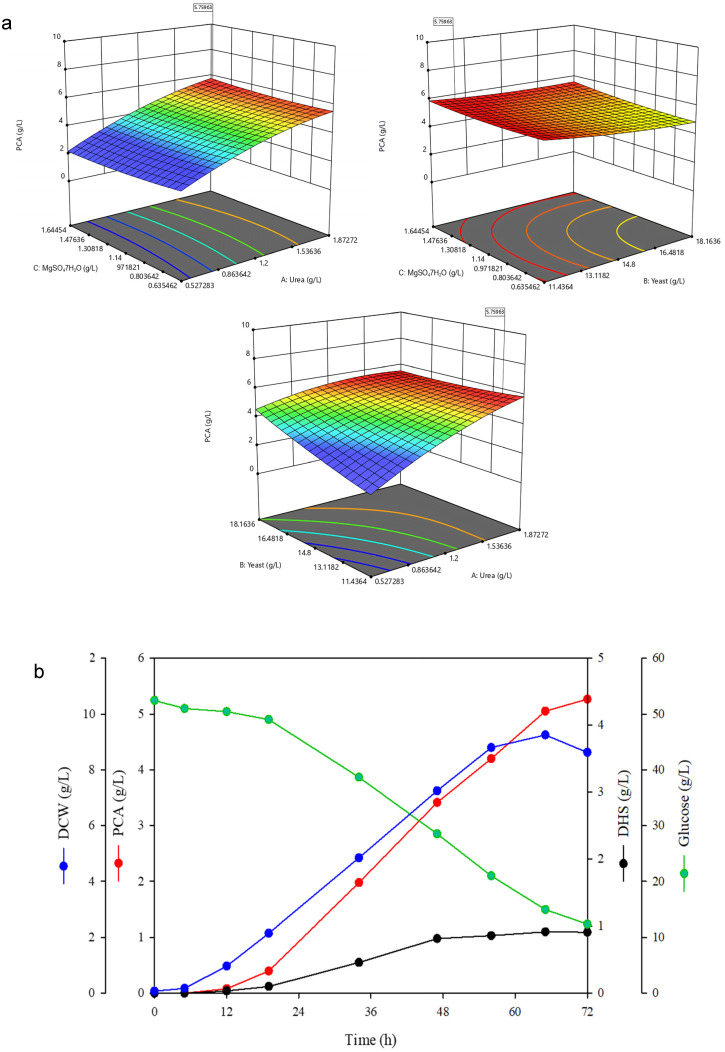
The RSM results and time-course profiles in 5L-batch cultures using CPM_2 production medium. (**a**) 3D response surface for PCA production of *C. glutamicum*. (**b**) Fermentation for medium optimization on PCA production in a 5 L fermenter. Fermentation was conducted for 72 h in batch culture by *C. glutamicum* AK103 with CPM_2. Error bars represent the standard deviation of two independent experiments. DCW, dry cell weight; PCA, protocatechuic acid; DHS, dehyroshikimic acid.

Based on the first-order model equation obtained from the FFD experiments above, the parameters for the steepest ascent method (SAM) experiments were calculated (Table S3) [41]. SAM is a strategy for approaching the region of maximal production based on the results of the FFD [33]. As shown in Table S3, SAM experiments were conducted in six steps, and the highest PCA production was observed in step 4. We designed the central composite design with three key factors (urea, yeast extract, and MgSO_4_⋅7H_2_O), which most affected PCA production based on the FFD (Table 2). As a result, three critical factors affecting PCA productivity can be logically selected through SAM, which enables efficient experimental design in the following response surface method (RSM) experiments. The RSM experimental design and the corresponding results are summarized in Table S4. The RSM experiments showed that the model was significant, with a *p*-value of 0.0159, and a quadratic polynomial was calculated to explain the influence and interaction of the medium components (Table 3). Statistical analysis of variance (ANOVA) demonstrated that the urea concentration exhibited highly significant effects (*p* < 0.001), establishing it as the primary control factor in the process optimization. The yeast extract also showed statistical significance (*p* < 0.05), albeit with less pronounced effects compared to urea. This finding indicates that the proportional relationship between urea and yeast extract concentrations in the production medium could play a substantial role in PCA production. In Figure 2a, the RSM results are shown in a three-dimensional model with the interactions between each medium composition and the productivity of PCA. The chosen medium composition was named CPM_2 and was comprised of 53 g/L glucose, 1.8 g/L urea, 12.5 g/L yeast extract, 6.6 g/L (NH_4_)_2_SO_4_, 1.6 g/L MgSO_4_ 7H_2_O, 0.6 g/L KH_2_PO_4_, and 0.01 g/L CaCl_2_ through the Design Expert statistical software analysis. To confirm the CPM_2 PCA production medium, flask cultures were established. In the flask cultures with CPM_1 containing 2 g/L of yeast extract, 0.26 g/L of PCA was produced. However, with the CPM_2 medium in flask fermentation, PCA production increased approximately 17-fold to 4.4 g/L. These results demonstrated that the series of media optimizations effectively increased PCA productivity. In addition, to determine whether the increased PCA production observed with CPM_2 was applicable at the fermenter scale, we conducted a 5 L fermenter culture fermentation. Batch culture using the CPM_2 medium for 72 h resulted in 5.27 g/L of PCA, a 5.27-fold increase in productivity compared to the initial 5 L fermenter fermentation using CPM_1 (Figure 2b). Despite identical media compositions, the fermenter cultivation exhibited higher production concentrations compared to the flask cultivation, which can be attributed to enhanced oxygen transfer capabilities in the bioreactor system [26,32]. Statistical medium optimization resulted in a remarkable improvement in the DCW of CPM_1, increasing from less than 1 g/L to over 8 g/L. This significant enhancement in growth performance suggests that cell growth inhibition was effectively mitigated through the serial medium optimization process. The optimization was further validated by increased PCA productivity per unit cell, and subsequent batch fermentation results confirmed the successful scalability of the optimized conditions.

### 2.3. Continuous Cultivation: Strategy to Address PCA Cytotoxicity

**Figure 3:**
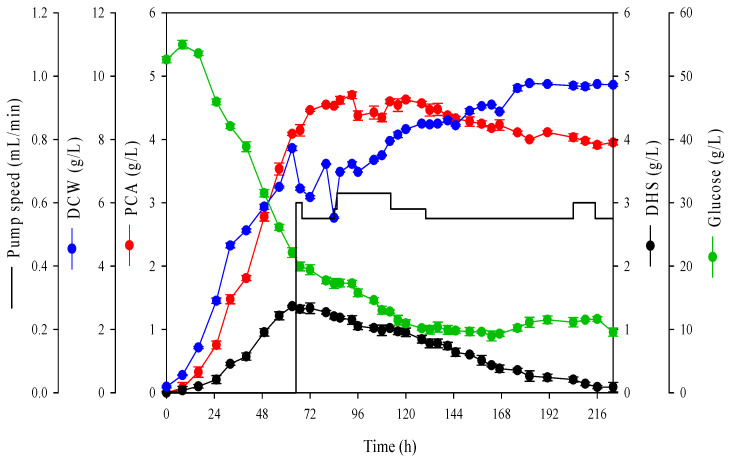
Effect of culture method on PCA production during fermentation in a 5 L fermenter. Fermentation was conducted for 224 h continuous culture by *C. glutamicum* AK104 with CPM_2 (63 h pump start; production medium: CPM_2; feeding: 2× CPM_2 medium). Error bars represent the standard deviation of two independent experiments. DCW, dry cell weight; PCA, protocatechuic acid; DHS, dehyroshikimic acid.

It is noteworthy that even under optimized media and fermentation conditions, PCA production exceeding 5 g/L was not achieved. Some researchers observed the cytotoxicity of PCA in *Corynebacterium* [16,17,19]. To confirm PCA cytotoxicity, we conducted gradient plate assays with PCA. For the gradient plates, 37 g/L of BHI agar was poured into a tilted plate left to solidify, and 10 g/L of PCA-containing BHI agar was added to a flat region of the plate (Figure S2). The cultivation results showed that regenerated colonies were only observed below about 6 g/L of the PCA concentration (Figure S2). Furthermore, in the bioreactor cultivations of *C. glutamicum* using the CPM_2 medium, we consistently observed that the cell growth rate decreased when the PCA accumulated to approximately 4–5 g/L in the medium. This suggests that growth inhibition due to PCA cytotoxicity occurs in both solid and liquid media cultivation. It is known that substrate depletion in batch cultivation can lead to decreased product formation [42]. To verify whether specific substrate depletion influences PCA production, fed-batch cultivation was conducted (Figure S3). The yeast extract solution and concentrated CPM_2 medium were used as feeding solutions. Similar to batch cultivation, we observed that PCA was not produced over 5 g/L in the fed-batch cultivation. This indicates that substrate depletion is not the primary cause of reduced PCA production. Therefore, we hypothesized that the production rate could be enhanced if we could lower the accumulated PCA concentration in the medium. To address the issue of reduced production rates owing to cell toxicity, we introduced a continuous culture process. Continuous cultures must be maintained at a steady state through a continuous supply of media. The steady state is defined as a constant concentration of cells, products, substrates, pH, and other environmental factors [43,44]. To establish the conditions for maintaining a steady state in continuous cultures, four preliminary experiments were conducted. The experiments involved adjusting the pump speed in the range of 0.1–0.91 mL/min to control dilution rate (*D)* and supplying the medium at various concentrations (1×, 2×, 4× and 8×) of CPM_2 to adjust the substrate supply rate (Figure S4). When 1× medium was supplied, the pump speed was adjusted to provide the necessary substrate for cell growth and PCA production. However, increasing the dilution rate to increase the substrate supply rate led to a washout of cells and an inability to maintain a steady state (Figure S3). This result is related to the condition that the specific growth rate (μ) must be equal to the dilution rate to maintain a steady state in continuous culture. Supplying 1.2× glucose and the remaining components at 2× as CPM_2 at a rate of 0.55 to 0.68 mL/min allowed the maintenance of a steady state. Under steady-state conditions, PCA remained at approximately 4 g/L, and glucose was maintained at approximately 10 g/L (Figure 3). In the steady state, where 4 g/L of PCA was reached, 0.064 g PCA/L h was produced from the elution rate of 16 mL/h of the actual medium, which was 78% higher than the productivity of batch culture. This study demonstrates that PCA accumulation produced by *C. glutamicum* has cytotoxic effects on cells, resulting in reduced productivity. By applying continuous culture to reduce the PCA concentration in the fermenter, this research is the first to successfully mitigate these cytotoxic effects.

## 3. Material and Methods

### 3.1. Strains, Media, and Plasmids

The *C. glutamicum* AK103 strain was derived from the wild-type strain ATCC 13032 [33]. Yeast extract and brain heart infusion (BHI) were purchased from Becton Dickinson (Franklin Lakes, NJ, USA). Glucose, ammonium sulfate, and sodium hydroxide were purchased from Duksan (Ansan, Republic of Korea). Potassium monophosphate and potassium diphosphate were purchased from Daejung Chemicals & Metals Co., Ltd. (Siheung, Republic of Korea). Hydrochloric acid, Antifoam 204, kanamycin sulfate, urea, magnesium sulfate, and calcium chloride were purchased from Sigma-Aldrich (St. Louis, MO, USA). The Masterflex^®^ L/S^®^ pump used for flow-through and continuous culture was purchased from Masterflex (Gelsenkirchen, Germany). *C. glutamicum* strains were grown in BHI medium, with the 48 h growth cultures in solid medium. The BHI was composed of calf brain infusion 7.7 g/L, beef heart infusion 9.8 g/L, proteose peptone 10 g/L, dextrose 2 g/L, sodium chloride 5 g/L, and disodium phosphate 2.5 g/L. Liquid cultures were grown by inoculating the colonies in BHI broth and performing a 15 h primary liquid culture at 30 °C and 200 rpm, followed by a 9 h secondary growth culture. The secondary growth culture was used as an inoculum for the PCA production cultures. The PCA production medium was inoculated with a secondary culture aliquot at 5% (*v*/*v*). After inoculation, the cultures were shaken for 72 h at 30 °C and 200 rpm. In addition, for the vector-inserted strains, 25 ppm kanamycin was added at all stages of culture.

### 3.2. Flask Cultures and 5 L Fermentation

For PCA production, a single colony was grown in 37 g/L of BHI agar at 30 °C for 16 h. The secondary culture was cultivated using 1% (*v*/*v*) of the inoculum in the same medium. The production culture was grown in a 300 mL flask, 220 rpm at 30 °C, with a working volume of 40 mL, for 3 days. For the production cultures, CPM_1 [50 g/L glucose; 2 g/L yeast extract; 5 g/L urea; 5 g/L ammonium sulfate; 0.5 g/L magnesium sulfate heptahydrate; 0.5 g/L potassium phosphate dibasic; 0.5 g/L potassium phosphate monobasic; and 0.01 g/L calcium chloride] production was used with the appropriate antibiotics. For a 5 L fermentation, the bottom-driven fermenter used for the production culture was KF-5 (Inchon, Republic of Korea), and the culture was grown in 3 L. Air was supplied at 1 bar and 1 *vv*m, the amount of dissolved oxygen was monitored with a DO probe (Mettler Toledo, Zurich, Switzerland), and the culture was agitated at 100–300 rpm. Antifoam was added (1 mL/L of production medium) to suppress foam formation. An Inpro^®^ 3030 (Mettler Toledo, Zurich, Switzerland) pH probe was used to keep the cultures within the optimal growth pH range (6.5–7.2) with 6 N HCl and 6 N NaOH. An inoculum dose of 5% was used for both the growth and production cultures. The fed-batch cultures were supplied with specific components or entire components of the production medium to provide the required limiting substrate (see Section 3).

### 3.3. Metabolite Analysis

The PCA and DHS were analyzed using a Waters W2695 HPLC system (34 Maple St., Milford, MA, USA). The samples collected during fermentation were diluted 100 times with distilled water, and the diluted fermentation broth was filtered through a syringe filter (20 μm pore size). HPLC analysis was performed on an HPX-87H column (300 × 7.8 hydrogen form, 9 µm particle size, 8% cross-linkage, Bio-Rad Laboratories, Hercules, CA, USA) with an injection volume of 10 μL and column heating at 50 °C. A 10 mM H_2_SO_4_ solution was used as the mobile phase, and the flow rate was maintained at 0.6 mL/min. A UV detector was used to analyze the *C. glutamicum* metabolome. The *C. glutamicum*, PCA, and DHS metabolites were detected and analyzed at 262 nm using the Waters W2998 HPLC system. The detector’s temperature was maintained at 40 °C during analysis. Standard curves were generated to observe the organic acid production and sugar consumption. To monitor cell growth, the OD_600_ was measured with a UV spectrophotometer (UV1800, Shimadzu, Kyoto, Japan). The optical density at 600 nm was converted to dry cell weight (DCW) using a correlation coefficient of 0.25 [45,46].

### 3.4. Statistical Optimization

Design Expert 12.0.3.0 software (Stat-Ease Inc., Minneapolis, MN, USA) was used for the experimental design and data analysis [37]. Statistical analysis of the model was performed to evaluate the ANOVA.

Prior to statistical optimization of the medium, we confirmed the effect of the yeast extract, a nitrogen source, on PCA production through the experiments shown in Figure S1. We set the center point for statistical optimization using 18 g/L of yeast extract, which showed the highest production, along with the remaining composition of CPM_1. A fractional factorial design was employed to screen for significant factors affecting the protocatechuic acid production and to adjust the non-significant factors in subsequent experiments. Using the six established factors, we utilized 2^5^ experimental points (Table S2). We employed fractional factorial design, which allows for the estimation of the main effects and some interactions while reducing the experimental scale to minimize errors. In FFD, factor settings were established with the center point as 0 and the high and low levels as +1 and −1, respectively. The results of the FFD are presented in Table 1.

The main effects were calculated through fractional factorial design, and coefficients representing the effect per unit were computed. Using these calculated indicators, we conducted the SAM. It was calculated using the equation $r=\sqrt{\sum (b_{i}2)}\;(b_{i}=Origin\;step)$, which yielded $Slope=\frac{r\times effect(X_{\left(1,2\ldots x\right)})}{\sqrt{\sum (b_{i}2)}\;}\;(b_{i}=main\;effect)$. Through slope values and origin step, we obtained new steps for creating experimental points and designed six SAM experiments using new step II values derived from our arbitrary coefficients. The results of these experiments can be found in Table S3.

We utilized a complete quadratic model, commonly used for analyzing curvature effects in response surface analysis, expressed as $Y=\beta_{0}\;+\;\Sigma \;\beta_{i}X_{i}\;+\;\Sigma \;\beta_{\mathrm{ii}}X_{i}2\;+\;\Sigma \;\beta_{\mathrm{ij}}X_{i}X_{j}$. Here, *Y* represents the response result, *β*_0_ is the intercept term serving as the reference point of the response surface, *β*_1_ represents the coefficients indicating linear effects, *β*_ii_ represents the coefficients indicating quadratic effects of each factor, and *β*_ij_ represents the coefficients indicating interaction effects between factors. Central composite design and response surface methodology were performed to optimize PCA production. In the response surface analysis experiments, we used composition 4 in Table S3, which showed the highest productivity in the SAM experiments as the center point. We established three highly influential factors and added axial points α to understand both linear and non-linear relationships, with α set at 1.68179 for three factors. The results of the RSM experiments can be found in Table 2 and Table 3. We used R^2^ values to assess how well our model explained the data, F-value to confirm the significance of the regression model, and *p*-value to evaluate the actual effects of individual factors and their interactions.

## 4. Conclusions

The PCA production process using *C. glutamicum* was improved using statistical media optimization and continuous culture processes. Statistical optimization of the production medium CPM_2 achieved a PCA production of 5.27 g/L, a 5.27-fold increase compared to that of CPM_1. However, PCA concentrations exceeding 5 g/L severely inhibited *C. glutamicum* PCA production. We, therefore, developed a continuous culture method that operated for 150 h in the steady-state mode. By reducing the PCA concentration in the production medium, PCA production continued without inhibiting cell growth. These findings suggest various options for improving PCA production and are expected to contribute to an efficient synthesis process. The improved PCA production process can reduce resource waste and environmental pollution while also helping to decrease dependence on chemical processes. Although this study achieved significant results through statistical optimization methods, future research could incorporate machine learning technologies like artificial neural networks and evolutionary algorithms for media optimization. These advanced techniques could reveal hidden correlations between variables that traditional statistical methods may miss, potentially leading to further improvements in PCA production using *C. glutamicum* at the industrial scale.

## Figures and Tables

**Table 1:** ANOVA generated using Design Expert software (Stat-Ease 12) for the two-level factorial design for optimized PCA production.

Analysis of Variance (ANOVA)					
Source	Sum of Squares	DF	Mean Square	F-Value	*p*-Value
Model	81.63	25	3.27	6.64	0.0128
A	1.7	1	1.7	3.45	0.1127
B	59.31	1	59.31	120.66	< 0.0001
C	0.2733	1	0.2733	0.5559	0.4841
D	0.5475	1	0.5475	1.11	0.3319
E	0.0323	1	0.0323	0.0658	0.8061
F	0.8839	1	0.8839	1.8	0.2285
AB	0.1042	1	0.1042	0.2119	0.6615
AC	1.66	1	1.66	3.37	0.116
AD	0.0988	1	0.0988	0.201	0.6696
AE	0.5691	1	0.5691	1.16	0.3233
AF	0.0161	1	0.0161	0.0327	0.8624
BC	1.64	1	1.64	3.33	0.1166
BD	1.36	1	1.36	2.77	0.1474
BE	0.1024	1	0.1024	0.2082	0.6642
BF	0.7674	1	0.7674	1.56	0.258
CD	0.0556	1	0.0556	0.1132	0.748
CE	0.6184	1	0.6184	1.26	0.3049
CF	0.0449	1	0.0449	0.0913	0.7727
DE	0.608	1	0.608	1.24	0.3087
DF	4.18	1	4.18	8.51	0.0267
EF	2.52	1	2.52	5.12	0.0642
Residual	2.95	6	0.4916		
Cor total	84.58	31			

A: glucose; B: urea; C: (NH_4_)_2_SO_4_; D: glucose; E: KH_2_PO_4_; F: MgSO_4_⋅7H_2_O. DF: degree of freedom.

**Table 2:** Coding and assigned concentration (g/L) of variables of different central composite design levels (alpha = 1.68). The concentrations of the other components in the production medium were set according to the conditions of step 4 in Table S3. Code levels −alpha/+alpha represent the axial points, −1/+1 the factorial points as reduced and elevated concentrations, and 0 is the center point of each factor.

Factor	Variable (g/L)	−Alpha (1.68)	−1	0	+1	+Alpha (1.68)
A	Urea	0.52	0.8	1.2	1.6	1.87
B	Yeast extract	11.43	12.8	14.8	16.8	18.16
C	MgSO_4_⋅7H_2_O	0.63	0.84	1.14	1.44	1.64

**Table 3:** ANOVA generated using Design Expert software (Stat-Ease 12) of the quadratic models for the central composite design (CCD) for optimized PCA production.

Analysis of Variance (ANOVA)					
Source	Sum of Squares	DF	Mean Square	F-Value	*p*-Value
Model	8.35	9	0.9276	5.7	0.0159
A-Urea	6.25	1	6.25	38.38	0.0004
B-Yeast extract	0.9814	1	0.9814	6.03	0.0438
C-MgSO_4_·7H_2_O	0.0316	1	0.0316	0.1939	0.673
AB	0.559	1	0.559	3.43	0.1063
AC	0.0189	1	0.0189	0.1163	0.743
BC	0.0211	1	0.0211	0.1299	0.7292
A^2^	0.2917	1	0.2917	1.79	0.2226
B^2^	0.0306	1	0.0306	0.1879	0.6777
C^2^	0.0291	1	0.0291	0.1789	0.685
Residual	1.14	7	0.1628		
Lack of Fit	1.13	5	0.2259	45.48	0.0217
Pure Error	0.0099	2	0.005		
Cor Total	9.49	16			

A: urea; B: yeast extract; C: MgSO_4_·7H_2_O. DF: degree of freedom.

## Data Availability

The data and materials generated or analyzed during this study are included in this published article and its Supplementary Information files.

## Supplementary Materials

The following supporting information can be downloaded at: https://www.mdpi.com/article/10.3390/ijms26010396/s1.

## Abbreviations

ANOVA: analysis of variance; BHI, brain heart infusion; DCW, dry cell weight; DHS, 3-dehydroshikimate; FFD, fractional factorial design; PCA, protocatechuate acid; RSM, response surface method; SAM, steepest ascent method.
