# Supplementary material for: Enhancement Strategy for Protocatechuic Acid Production Using Corynebacterium glutamicum with Focus on Continuous Fermentation Scale-Up and Cytotoxicity Management
Source: Int J Mol Sci. 2025 Jan 5;26(1):396. doi: 10.3390/ijms26010396 (PMC11720217; doi:10.3390/ijms26010396)
Supplement: Supplementary file 1 [file ijms-26-00396-s001.zip › ijms-3380489-supplementary.docx]

**Enhancement Strategy for Protocatechuic Acid Production Using *Corynebacterium glutamicum* with Focus on Continuous Fermentation Scale-Up and Cytotoxicity Management**

Jiwoon Chung^1,2†^, Woo-Shik Shin^2†^, Chulhwan Park^1,^* and Jaehoon Cho^2,^*

*** Corresponding authors:**

Chulhwan Park, PhD

Department of Chemical Engineering

Kwangwoon University

20, Gwangun-ro, Nowon-gu, Seoul, 01897, Republic of Korea

Tel: +82-2-940-5173

E-mail address: chpark@kw.ac.kr

Jaehoon CHO, PhD

Green and Sustainable Materials R&D Department

Korea Institute of Industrial Technology (KITECH)

89, Yangdaegiro-gil, Ipjang-myeon, Seobuk-gu, Cheonan, 31056, Republic of Korea

Tel: +82-41-589-8341

E-mail address: cjh0107@kitech.re.kr

^†^ These authors contributed equally to this work

**Supporting Information**


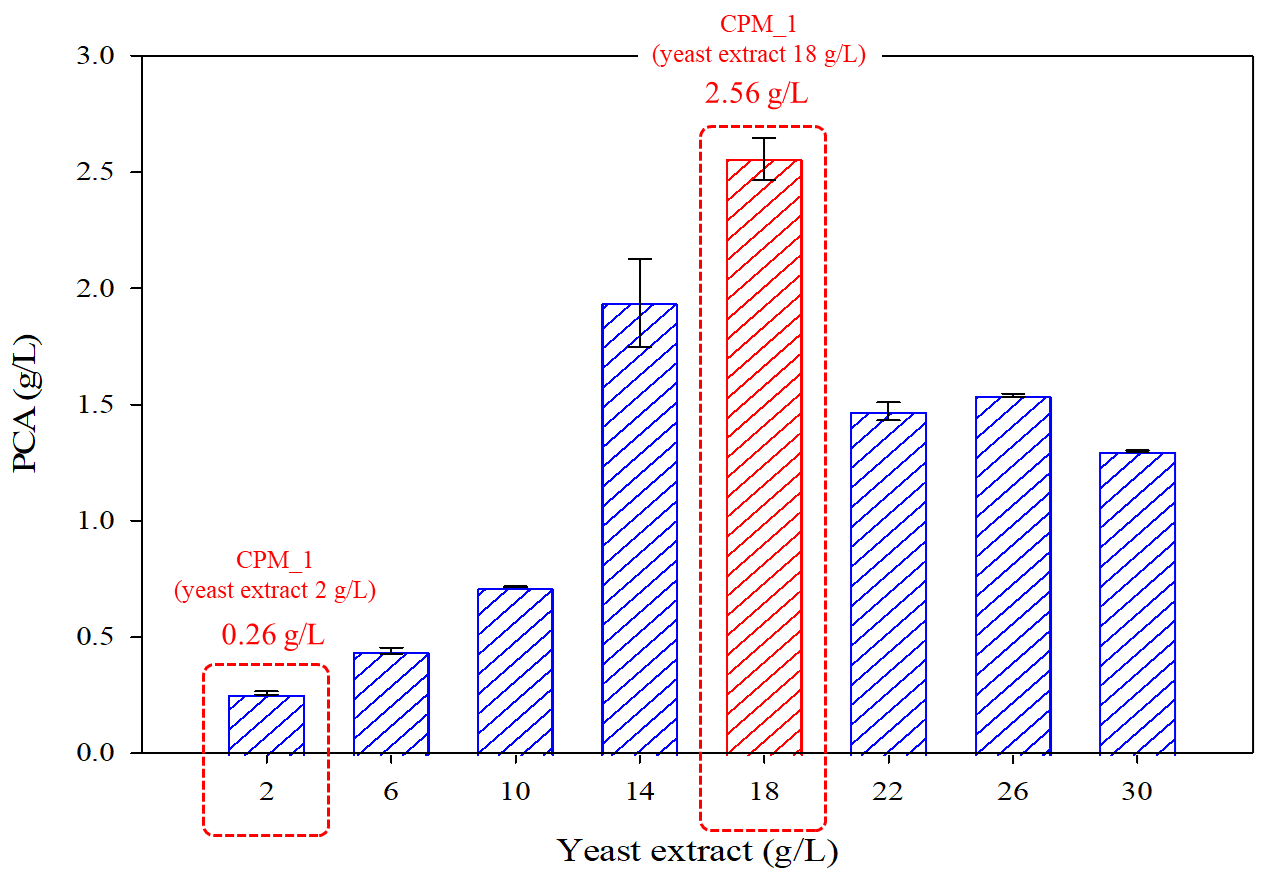


Figure. S1. Effect of yeast extract on PCA production during fermentation in flask cultures. Fermentation was conducted for 72 h in batch culture by *C. glutamicum* AK103 with CPM_1.

Table S1. Coding and assigned concentration (g/L) of variables of different levels for two-level factorial design. Code levels represent a change in concentration of each factor: -1, reduced concentration; +1, elevated concentration; 0, center point.

| **Factor** | **Variable** | **-1** | **0** | **1** |
| --- | --- | --- | --- | --- |
| A | Glucose | 35 | 50 | 65 |
| B | Urea | 2 | 5 | 8 |
| C | (NH_4_) _2_SO_4_ | 2 | 5 | 8 |
| E | Yeast extract | 14 | 18 | 22 |
| E | KH_2_PO_4_ | 0 | 0.5 | 1 |
| F | MgSO_4_⋅7H_2_O_4_ | 0 | 0.5 | 1 |

Table S2. Experimental matrix for the fractional factorial design. A, glucose; B, urea; C, (NH_4_)_2_SO_4_; D, yeast extract; E, KH_2_PO_4_; F, MgSO_4_⋅7H_2_O.

| **Run** | **A: Glucose** | **B: Urea** | **C:** (NH_4_)_2_SO_4_ | **D: Yeast extract** | **E: KH_2_PO_4_** | **F:** MgSO_4_⋅7H_2_O | **Response PCA g/L** |
| --- | --- | --- | --- | --- | --- | --- | --- |
| 1 | 65 | 8 | 8 | 22 | 0 | 0 | 2.66 |
| 2 | 65 | 2 | 8 | 22 | 1 | 1 | 3.83 |
| 3 | 65 | 2 | 2 | 14 | 0 | 1 | 5.63 |
| 4 | 65 | 2 | 2 | 14 | 1 | 0 | 0.98 |
| 5 | 65 | 8 | 2 | 22 | 1 | 0 | 2.78 |
| 6 | 35 | 8 | 2 | 22 | 0 | 0 | 2.02 |
| 7 | 65 | 8 | 8 | 14 | 1 | 0 | 1.10 |
| 8 | 35 | 2 | 8 | 14 | 0 | 1 | 4.12 |
| 9 | 65 | 2 | 8 | 14 | 0 | 0 | 5.43 |
| 10 | 35 | 8 | 2 | 14 | 1 | 0 | 0.93 |
| 11 | 65 | 8 | 2 | 14 | 1 | 1 | 1.99 |
| 12 | 65 | 8 | 2 | 14 | 0 | 0 | 2.40 |
| 13 | 65 | 2 | 2 | 22 | 1 | 1 | 4.62 |
| 14 | 35 | 8 | 8 | 22 | 1 | 0 | 1.54 |
| 15 | 35 | 2 | 2 | 22 | 1 | 1 | 3.68 |
| 16 | 65 | 2 | 2 | 22 | 0 | 0 | 4.26 |
| 17 | 65 | 2 | 8 | 22 | 0 | 0 | 4.75 |
| 18 | 65 | 2 | 8 | 22 | 1 | 0 | 4.57 |
| 19 | 65 | 2 | 8 | 14 | 1 | 1 | 6.80 |
| 20 | 35 | 8 | 2 | 22 | 1 | 0 | 2.13 |
| 21 | 35 | 8 | 2 | 14 | 0 | 1 | 2.34 |
| 22 | 65 | 8 | 8 | 14 | 0 | 1 | 1.96 |
| 23 | 35 | 2 | 2 | 14 | 1 | 1 | 5.12 |
| 24 | 35 | 8 | 8 | 22 | 0 | 1 | 2.02 |
| 25 | 65 | 8 | 2 | 22 | 0 | 1 | 0.45 |
| 26 | 35 | 2 | 2 | 22 | 0 | 1 | 3.47 |
| 27 | 65 | 8 | 8 | 22 | 1 | 1 | 2.03 |
| 28 | 35 | 8 | 8 | 14 | 1 | 1 | 2.19 |
| 29 | 35 | 8 | 8 | 14 | 0 | 0 | 0.47 |
| 30 | 35 | 2 | 8 | 22 | 0 | 0 | 3.89 |
| 31 | 35 | 2 | 2 | 14 | 0 | 0 | 4.42 |
| 32 | 35 | 2 | 8 | 14 | 1 | 0 | 4.94 |

Table S3. Experiment based on the center point of FFD to perform ascent method (SAM). Parameters for the SAM experiment (r value, slope value, new step, and new step Ⅱ) were calculated based on the factorial design.

|  | | Glucose | | Urea | | (NH_4_)_2_SO_4_ | | Yeast extract | | KH_2_PO_4_ | | MgSO_4_⋅7H_2_O | |  |
| --- | --- | --- | --- | --- | --- | --- | --- | --- | --- | --- | --- | --- | --- | --- |
| Center point | | 50 | | 5 | | 5 | | 18 | | 0.5 | | 0.5 | |  |
| Origin step | | 15 | | 3 | | 3 | | 4 | | 0.5 | | 0.5 | |  |
| Main effect | | 0.23 | | -1.36 | | 0.096 | | -0.13 | | 0.03 | | 0.16 | |  |
| Coefficient | | 0.115 | | -0.68 | | 0.048 | | -0.065 | | 0.015 | | 0.08 | |  |
|  | |  | |  | |  | |  | |  | |  | |  |
| r Value | | 1.6109004 | |  | |  | |  | |  | |  | |  |
| Slope value | | 0.2649783 | | -1.588465 | | 0.674188 | | -1.005234 | | 0.2968705 | | 1.6109004 | |  |
| New step | | 3.974674 | | -4.765395 | | 2.0225639 | | -4.020936 | | 0.1484352 | | 0.8054502 | |  |
| New step II | | 0.7949348 | | -0.953079 | | 0.4045128 | | -0.804187 | | 0.029687 | | 0.16109 | |  |
|  | |  | |  | |  | |  | |  | |  | |  |
| Steps | Glucose | | Urea | | (NH_4_)_2_SO_4_ | | Yeast extract | | KH_2_PO_4_ | | MgSO_4_⋅7H_2_O | | PCA (g/L) | |
| Center point | 50 | | 5 | | 5 | | 18 | | 0.5 | | 0.5 | |  | |
| 1 | 50.794 | | 4.05 | | 5.4 | | 17.2 | | 0.52 | | 0.66 | | 1.95 | |
| 2 | 51.588 | | 3.1 | | 5.8 | | 16.4 | | 0.54 | | 0.82 | | 2.55 | |
| 3 | 52.382 | | 2.15 | | 6.2 | | 15.6 | | 0.56 | | 0.98 | | 3.01 | |
| 4 | 53.176 | | 1.2 | | 6.6 | | 14.8 | | 0.58 | | 1.14 | | 3.31 | |
| 5 | 53.97 | | 0.25 | | 7 | | 14 | | 0.6 | | 1.3 | | 3.1 | |
| 6 | 54.764 | | 0 | | 7.4 | | 13.2 | | 0.62 | | 1.46 | | 1.75 | |

Table S4. Experimental matrix of the central composite design (CCD) and PCA production for each experiment. Factor A, urea; Factor B, yeast extract; Factor C, MgSO_4_⋅7H_2_O.

| Run | Factor A: UREA | Factor B: Yeast extract | Factor C: MgSO_4_⋅ 7H_2_O | Response 1: PCA g/L |
| --- | --- | --- | --- | --- |
| 1 | 1.2 | 11.43 | 1.14 | 3.79 |
| 2 | 0.8 | 16.8 | 1.44 | 4.15 |
| 3 | 1.2 | 14.8 | 1.14 | 4.64 |
| 4 | 0.8 | 12.8 | 1.44 | 3.25 |
| 5 | 1.2 | 14.8 | 1.14 | 4.58 |
| 6 | 1.87 | 14.8 | 1.14 | 4.73 |
| 7 | 1.2 | 14.8 | 1.14 | 4.39 |
| 8 | 1.6 | 16.8 | 0.84 | 5.20 |
| 9 | 1.6 | 12.8 | 0.84 | 5.56 |
| 10 | 1.6 | 12.8 | 1.44 | 5.58 |
| 11 | 0.8 | 12.8 | 0.84 | 3.47 |
| 12 | 1.2 | 14.8 | 1.64 | 4.77 |
| 13 | 1.6 | 16.8 | 1.44 | 5.38 |
| 14 | 0.8 | 16.8 | 0.84 | 4.11 |
| 15 | 1.2 | 18.16 | 1.14 | 5.38 |
| 16 | 0.52 | 14.8 | 1.14 | 3.24 |
| 17 | 1.2 | 14.8 | 0.63 | 4.39 |

a


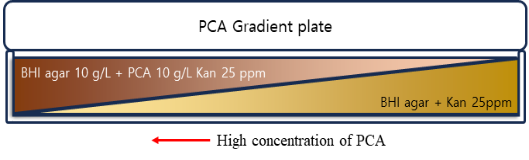


b


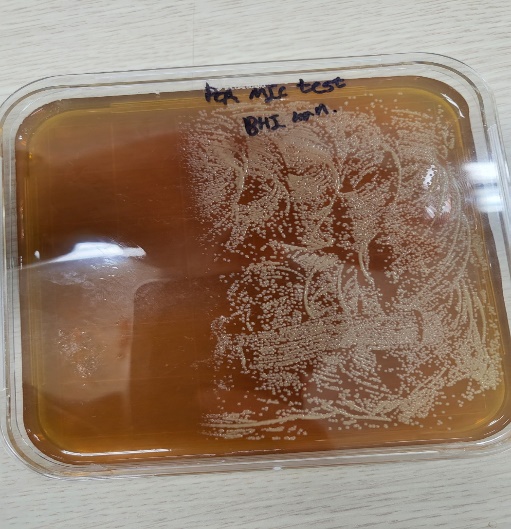


Figure. S2. PCA gradient agar plate. (a) Schematic diagram of a PCA gradient agar plate. (b) Photograph of growing strain colonies. To create a concentration gradient, square plant culture dishes were used because of their convenience and larger surface area for the solid agar culture. The cultured cells were then spread onto the gradient plate using the 100 μL spreading method and incubated for 48–72 h.

a

b

**
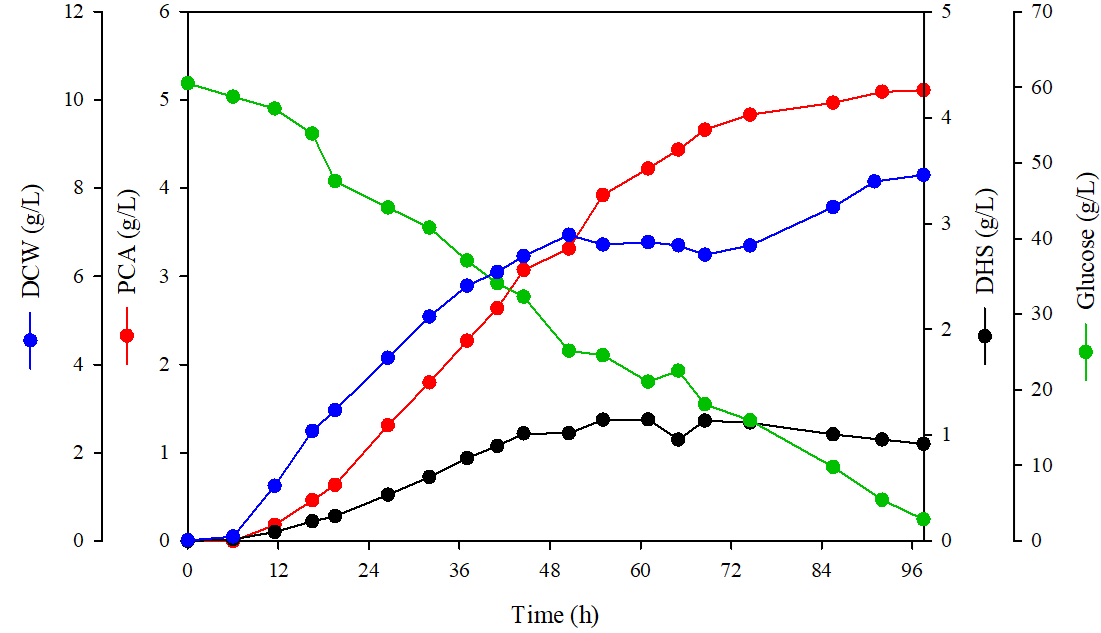
**

Figure. S3. Effect of substrate supply methods on PCA production during fermentation in a 5 L fermenter. (a) Fermentation was conducted for 97.5 h in fed-batch culture by *C. glutamicum* AK103 with CPM_2 (46 h feeding start; pulse-feeding of 100 g/L yeast extract). (b) Fermentation was conducted for 97.5 h in fed-batch culture by *C. glutamicum* AK104 with CPM_2 (46 h feeding start; pulse-feeding of 8× CPM_2).

a b


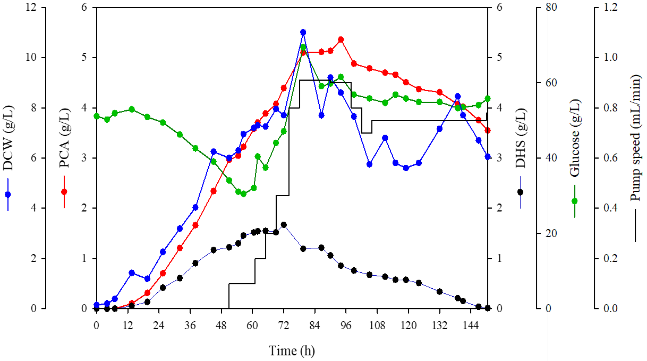


c d

Figure. S4. Time-course profiles of cell growth, sugar consumption, pump speed, and metabolite production in continuous cultures. (a) Fermentation was conducted for 150.5 h in continuous culture with CPM_2 (51 h pump start; production medium: CPM_2; feeding: 8× CPM_2 medium (51~96 h); 1× CPM_2 medium (96 h~)). The 5 L fermentation was performed under the operating conditions. (b) Fermentation was conducted for 150.5 h in continuous culture with CPM_2 (51 h pump start; production medium: CPM_2; feeding: 8× CPM_2 medium (51~80 h); 2× CPM_2 medium (80 h~)). (c) Fermentation was conducted for 172 h in continuous culture by *C. glutamicum* AK104 with CPM_2 (65 h pump start; production medium: CPM_2; feeding: 1× CPM_2 medium). (d) Fermentation was conducted for 191 h in continuous culture by *C. glutamicum* AK104 with CPM_2 (65 h pump start; production medium: CPM_2; feeding: 4× CPM_2 medium).
